# A proteome-wide association study reveals novel plasma proteins as potential therapeutic targets for metabolic dysfunction-associated steatotic liver disease

**DOI:** 10.3389/fendo.2025.1664691

**Published:** 2025-10-16

**Authors:** Qian Zhong, Yue Zhu, Yiwen Tan, Wenzeng Deng, Chunxia Wang, Shan Yang, Nanwei Tong

**Affiliations:** ^1^ Department of Endocrinology, West China Hospital, Sichuan University, Chengdu, China; ^2^ Department of Operating Room, West China Hospital, West China School of Nursing, Sichuan University, Chengdu, China; ^3^ Department of Pathology, The Second Affiliated Hospital, Chongqing Medical University, Chongqing, China; ^4^ Department of Endocrinology, Chongqing University Qianjiang Hospital, Chongqing, China; ^5^ Department of Nephrology, The Second Affiliated Hospital, Chongqing Medical University, Chongqing, China; ^6^ Laboratory of Diabetes and Metabolism Research, West China Hospital, Sichuan University, Chengdu, China

**Keywords:** MASLD, proteome-wide association study, Mendelian randomization, NCAN, liver diseases, genetics

## Abstract

**Introduction:**

Metabolic dysfunction-associated steatotic liver disease (MASLD) is a growing global health burden with limited therapeutic options. To identify novel proteins involved in its pathogenesis and reveal potential drug targets, we performed an integrative analysis combining plasma proteomic data with genome-wide association study (GWAS) summary statistics for MASLD.

**Methods:**

A proteome-wide association study (PWAS) was conducted by integrating plasma protein quantitative trait loci (pQTL) data with GWAS summary statistics from the FinnGen R11 MASLD cohort (used as the discovery dataset) and a large-scale MASLD GWAS meta-analysis (used for validation). Causal inference was assessed using Mendelian Randomization (MR), and Bayesian colocalization was applied to identify shared genetic signals. Additionally, liver specimens from five healthy controls and five MASLD patients were subjected to H&E and NCAN immunohistochemistry.

**Results:**

PWAS in the discovery cohort identified three plasma proteins—NCAN, EPHA2, and APOE—significantly associated with MASLD risk. Among them, NCAN showed the strongest and most consistent association, which was replicated in the validation cohort. MR analyses supported a causal role for NCAN in both cohorts, whereas colocalization at the *NCAN* locus was suggestive rather than definitive. Immunohistochemical analysis showed that *NCAN* expression was significantly reduced in MASLD liver tissues.

**Conclusions:**

This integrative proteomic and genetic study identified NCAN as a key contributor to MASLD pathogenesis. Its consistent association and genetic evidence across two independent cohorts highlight NCAN as a promising therapeutic target that merits further functional investigation.

## 1 Introduction

Metabolic dysfunction-associated steatotic liver disease (MASLD) is defined as a metabolic disorder characterized primarily by the accumulation of lipids within hepatocytes, in the absence of excessive alcohol consumption or other known causes of liver injury (1). MASLD has become one of the most prevalent chronic liver diseases worldwide, with a markedly increasing incidence in recent years (2). According to the World Health Organization, the global prevalence of MASLD has exceeded 30%, and this figure is significantly higher among individuals with obesity and diabetes (3). Historically, this condition was referred to as nonalcoholic fatty liver disease (NAFLD), first described by Ludwig et al. in 1980 as a liver disorder mimicking alcoholic hepatitis that occurred in individuals with obesity and other metabolic comorbidities but no significant alcohol intake (4). Over the years, the limitations of the exclusionary definition of NAFLD, along with its close links to metabolic risk factors, have prompted efforts to redefine this entity (5). In 2020, the term metabolic dysfunction-associated fatty liver disease (MAFLD) was proposed, followed by the international adoption of MASLD in 2023 as part of a multi-society Delphi consensus, recognizing the metabolic drivers of this condition and officially retiring the term NAFLD (6). The pathogenesis of MASLD is closely associated with hepatic lipid metabolism dysregulation, insulin resistance, oxidative stress, and chronic inflammation (7). As the disease progresses, patients may develop metabolic dysfunction associated steatohepatitis (MASH), fibrosis, and ultimately cirrhosis or hepatocellular carcinoma (8). Therefore, early identification and intervention are of critical importance.

Over the past decade, genome-wide association studies (GWAS) have significantly advanced our understanding of the genetic architecture underlying MASLD (9). A substantial proportion of the associated variants are located in non-coding genomic regions and are believed to exert their effects by modulating gene expression and protein production (10). Despite these discoveries, the biological pathways that connect most genetic loci to MASLD pathogenesis remain largely undefined. To date, only a limited number of loci—such as *PNPLA3*, *TM6SF2*, and *HSD17B13*—have been functionally validated in the context of MASLD (10). Furthermore, the identification of true causal variants is complicated by linkage disequilibrium, which often masks the individual contributions of specific polymorphisms (11). This gap between genetic association and biological function poses a major hurdle for clinical translation, particularly in developing gene-targeted therapies (12). Since proteins represent the downstream effectors of gene expression and are directly involved in disease processes, especially in MASLD, where circulating proteins play central roles, the plasma proteome offers a promising avenue for mechanistic insights and therapeutic intervention (13, 14). MASLD frequently disrupts plasma protein profiles, and these proteins are considered highly accessible targets for pharmacological modulation (15). Recent advancements in high-throughput proteomic technologies have enabled the identification of protein quantitative trait loci (pQTLs), which establish direct links between genetic variants and plasma protein levels (16). Integrating pQTL data with GWAS through proteome-wide association studies (PWAS) offers a comprehensive framework to uncover novel associations between the plasma proteome and MASLD, potentially revealing new molecular targets for treatment (17).

Uncovering effective therapeutic targets is essential for advancing treatment strategies and improving clinical outcomes in patients with MASLD. To systematically identify candidate drug targets for MASLD, we conducted a PWAS of 1,345 circulating plasma proteins using genetic data from the FinnGen R11 cohort (hereafter referred to as R11 MASLD) (18), and validated the findings in an independent MASLD dataset derived from the largest GWAS meta-analysis to date (19). To assess potential causality, we extracted *cis*-pQTLs for the identified proteins and applied Mendelian Randomization (MR) analysis. To evaluate whether the same causal variants underlie both protein abundance and MASLD risk, we performed Bayesian colocalization analyses for the discovery R11 MASLD cohort and the validation MASLD cohort. Collectively, these integrative analyses aim to pinpoint novel plasma proteins that are functionally linked to MASLD pathogenesis and offer promising avenues for drug development.

## 2 Materials and methods

### 2.1 Human plasma proteomic and genetic data

The human plasma proteomic data used for the PWAS were derived from the Atherosclerosis Risk in Communities (ARIC) cohort, specifically from plasma samples collected during the third study visit (17). This cohort includes individuals of both European and African American ancestry across various regions in the United States. To minimize confounding effects due to population stratification, the current analysis was restricted to participants of European descent. After excluding individuals lacking genotype information, 7,213 European-ancestry participants were retained for downstream analysis. Plasma protein quantification was conducted using the SOMAmer (slow off-rate modified aptamer) platform, a high-throughput proteomics technique that utilizes specific DNA-based aptamers to bind target proteins (20). In total, 4,657 SOMAmer reagents targeting 4,483 distinct proteins were measured in the original dataset (17).

Genotyping of the included individuals was performed using the Affymetrix 6.0 microarray platform. To identify *cis*-pQTLs, linear regression analyses were conducted with adjustment for key covariates, including age, sex, study center, ten principal components of genetic ancestry, and probabilistic estimation of expression residuals factors. The *cis*-regions for each protein-coding gene were defined as the genomic region spanning 500 kilobases upstream and downstream of the transcription start site. A total of 6,181,856 single nucleotide polymorphisms (SNPs) with minor allele frequency (MAF) > 1% within these regions were evaluated. Ultimately, 2,004 SOMAmers were found to have at least one statistically significant *cis*-pQTL (false discovery rate [FDR] < 5%) located near the gene encoding the corresponding protein.

### 2.2 GWAS data of MASLD

R11 MASLD statistics were obtained from the FinnGen R11 dataset (https://r11.finngen.fi/pheno/NAFLD), comprising 3,006 individuals diagnosed with MASLD and 450,727 control subjects. In the FinnGen dataset, MASLD was defined as hepatic steatosis not attributable to alcohol consumption. MASLD cases were identified using ICD-10 code K76.0, which was recorded either at hospital discharge or as the primary cause of death. Individuals lacking this diagnostic code were classified as controls. To minimize misclassification, individuals with alcoholic liver disease codes (ICD-10 K70.) were excluded, and MASH codes (ICD-10 K75.81) were not used to ascertain cases.

We additionally accessed summary statistics from the largest GWAS meta-analysis of MASLD (19) to date, which analyzed 8,434 individuals diagnosed with MASLD and 770,180 control participants, all of European ancestry. This analysis incorporated data from four large-scale cohorts: the Electronic Medical Records and Genomics (eMERGE) network, UK Biobank, FinnGen, and the Estonian Biobank. All contributing cohorts applied study-specific genotyping, imputation, and quality control, and phenotype definitions were standardized within each electronic health record environment. Institutional approvals and informed consent procedures followed the original publications. Because only summary statistics were available, no additional clinical, laboratory, imaging, or histologic criteria beyond billing codes could be uniformly applied across cohorts.

### 2.3 Proteome-wide association studies

**Figure 1:**
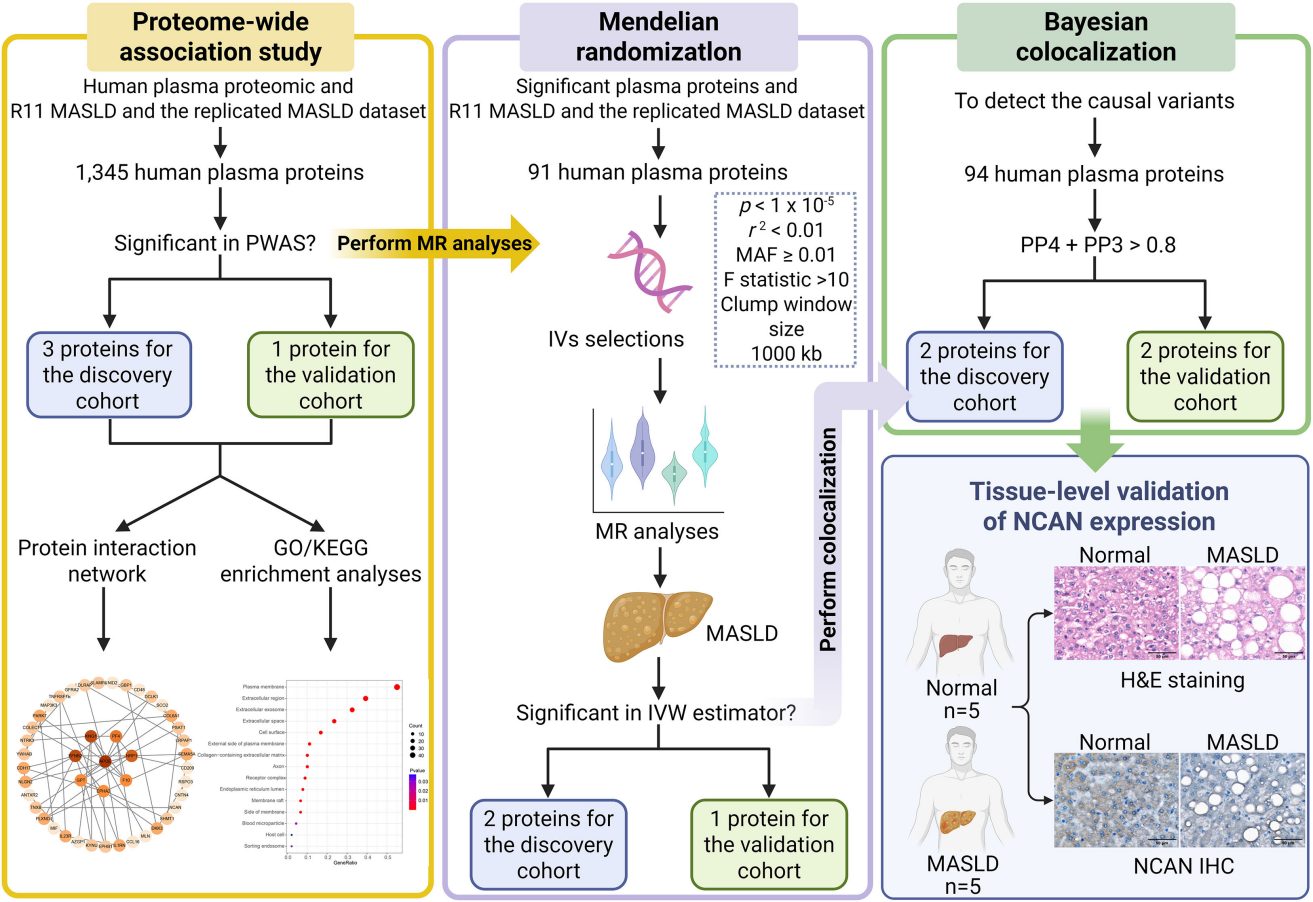
Summary of research framework and analytical approach. The study commenced with PWAS, utilizing the FUSION platform to explore the genetic links between *cis*-regulated circulating protein levels and the risk of MASLD, using both the FinnGen R11 dataset and the meta-analyzed MASLD GWAS dataset for discovery and validation, respectively. Subsequently, genes identified through PWAS were subjected to protein-protein interaction (PPI) network construction and functional enrichment analysis to uncover potential biological pathways. To move from association to causation, we applied MR to the significant proteins discovered in the PWAS phase. SNPs that fulfilled instrumental variable (IV) assumptions—such as relevance, independence, and exclusivity—were selected and evaluated for robustness through sensitivity testing, heterogeneity assessment, and pleiotropy diagnostics. Both the inverse variance weighted (IVW) method and MR-Egger regression were employed for causal inference. Furthermore, to investigate whether shared causal variants underlie changes in both protein abundance and MASLD susceptibility, Bayesian colocalization analysis was conducted on *cis*-pQTLs in both the discovery cohort (R11 MASLD) and the validation cohort (replicated MASLD GWAS dataset). Finally, tissue-level validation was conducted by H&E staining and NCAN immunohistochemistry using liver specimens from healthy controls (n=5) and MASLD patients (n=5). PWAS: proteome-wide association study; MASLD: metabolic dysfunction-associated steatotic liver disease; MR: Mendelian Randomization; IV: instrumental variable; MAF: minor allele frequency; H&E: hematoxylin and eosin staining; IHC: immunohistochemistry.

Proteins, as the final products of gene transcription, play central roles in the initiation and progression of MASLD. To systematically evaluate the relationship between circulating proteins and MASLD, we applied PWAS, conducted via the FUSION framework (http://gusevlab.org/projects/fusion), as illustrated in **Figure 1**. Initially, the SNP-based heritability for 2,004 SOMAmer protein measurements was calculated using the restricted maximum likelihood REML approach implemented in the GCTA software package (21). Among them, 1,345 proteins demonstrated significant *cis*-heritability (*P* < 0.01), indicating a genetic basis for plasma abundance variation. Next, FUSION was utilized to model the influence of SNPs on protein levels, employing both top1 and elastic net (enet) modeling strategies. The optimal model for each protein was selected based on its predictive accuracy for protein expression. These models were then used to integrate summary-level genetic associations from GWAS datasets of R11 MASLD and the broader MASLD cohort, combining Z-scores of SNP associations with protein prediction weights across loci to conduct the PWAS (22). Multiple testing correction was performed using the Benjamini-Hochberg procedure, and associations were considered statistically significant if the adjusted *p*-value was less than 0.05.

### 2.4 Protein interaction network and functional enrichment analyses

To gain deeper insight into the biological relevance of the proteins identified in the PWAS, we conducted PPI network analysis. Functional associations among these proteins were retrieved using the STRING database (https://string-db.org), with interaction pairs exhibiting a combined confidence score above 0.4 considered to be statistically meaningful. The resulting interaction network was visualized using Cytoscape (version 3.10.3). To detect densely interconnected protein clusters within the network, we applied the Molecular Complex Detection (MCODE) plugin in Cytoscape using default parameters. Key regulatory proteins, or hub genes, were prioritized based on network centrality measures, specifically betweenness, utilizing the cytoHubba algorithm.

Additionally, we used the DAVID online platform (https://davidbioinformatics.nih.gov/summary.jsp) to perform Gene Ontology (GO) and Kyoto Encyclopedia of Genes and Genomes (KEGG) enrichment analysis on proteins significantly associated with the phenotype (*p* < 0.05). The GO framework, commonly utilized in bioinformatics, was applied to classify gene products into three main categories: biological processes (BP), molecular functions (MF), and cellular components (CC). To explore potential biological pathways, KEGG analysis was employed, offering curated molecular datasets that facilitate the identification of relevant signaling and metabolic pathways based on gene enrichment. Visualization of enrichment results was conducted using the “ggplot2” package in R (version 4.2.1). Statistical significance was defined as a *p*-value less than 0.05.

### 2.5 Mendelian randomization analyses

To further validate the potential causal links between plasma proteins and MASLD susceptibility, we conducted MR analyses utilizing SNPs associated with significant proteins identified from the PWAS as IVs, applied separately to the discovery cohort and the validation cohort, as outlined in **Figure 1**. To ensure sufficient statistical power and include an adequate number of variants, the threshold for SNP inclusion was relaxed to a *p*-value less than 1 × 10^-5^. We also applied clumping procedures using a 1 Mb window, retaining only independent variants by excluding those in linkage disequilibrium (r^2^ ≥ 0.01) (23). To minimize weak instrument bias, the strength of the selected SNPs was assessed by calculating F-statistics, using the formula: F = R^2^ × (N − k − 1)/[(1 – R^2^) × k], where R^2^ indicates the proportion of variance explained. An F-statistic greater than 10 is generally considered acceptable (24), and in our study, all selected IVs had F-statistics exceeding 20, indicating robust instrument strength. To estimate the genetic effect of plasma proteins (exposures) on MASLD risk (outcomes), we applied the Wald ratio method for single-SNP IVs and inverse variance weighted (IVW) regression for multiple IVs, conducting separate analyses for the discovery dataset and the validation dataset. Additionally, MR-Egger regression was used as a sensitivity analysis to detect directional pleiotropy, with the intercept serving as an indicator—deviation from zero suggesting potential horizontal pleiotropic effects. Heterogeneity among IVs was evaluated using the Q-statistic from the IVW model. Leave-one-out analysis was also performed to assess whether any individual SNP unduly influenced the MR results. All statistical procedures were executed using established MR analysis packages in R, including “TwoSampleMR,” and “MendelianRandomization.” Correction for multiple comparisons was applied using the Benjamini-Hochberg procedure, and an adjusted *p*-value less than 0.05 was considered statistically significant. Only *cis*-pQTL instruments (± 1 Mb) were used; no trans IVs were included. The ARIC pQTL cohort and MASLD GWAS sources were assembled independently, with no intentional sample overlap; any inadvertent overlap is expected to be minimal.

### 2.6 Bayesian colocalization analyses

To evaluate whether a single genetic variant could simultaneously influence protein levels and MASLD risk, we implemented Bayesian colocalization analysis separately for two GWAS datasets: the discovery cohort and the validation cohort. Analyses were performed using the R package “coloc” (version 4.2.1) under default prior settings (p1 = 1 × 10^-4^; p2 = 1 × 10^-4^; p12 = 1 × 10^-5^) (25). In this context, p1 denotes the prior probability that a given variant is linked to MASLD; p2 refers to the prior for protein-related associations; and p12 reflects the prior probability that the same variant is involved in both phenotypes. Using GWAS summary statistics, the Approximate Bayes Factor was computed to generate posterior probabilities (PP) for five distinct hypotheses: H0: the variant has no effect on either trait (PP0); H1: the variant is associated only with MASLD (PP1); H2: the variant is only linked to protein expression (PP2); H3: two different variants independently affect the protein and disease traits (PP3); H4: a single variant exerts shared influence on both protein levels and disease-related phenotypes (PP4). In this study, we defined colocalization solely by PP4: signals with PP4 ≥ 0.8 were designated primary (colocalized) protein targets. Signals with 0.5 ≤ PP4 < 0.8 were labeled secondary (suggestive), prioritized for follow-up and not used to support causal or therapeutic claims. All others were considered tertiary (not colocalized/low priority) (26). To safeguard against low power, we used PP3+PP4 ≥ 0.8 only as a screening indicator to flag regions where both traits show association (27), but classification was based on PP4 alone.

### 2.7 H&E staining and immunohistochemistry

To further assess NCAN expression in liver tissues, five liver specimens from healthy controls and five pathologically confirmed MASLD patients were collected from Qianjiang Central Hospital of Chongqing. All procedures were approved by the hospital’s Institutional Ethics Committee (Approval No. QJZXYY-2025-008), and written informed consent was obtained from all participants. Tissue samples were fixed in 4% paraformaldehyde, routinely embedded in paraffin, sectioned at 4-µm thickness, and subjected to hematoxylin and eosin (H&E) staining and immunohistochemistry (IHC).

For IHC, the sections were deparaffinized in xylene, rehydrated through a graded ethanol series, and subjected to heat-induced antigen retrieval in citrate buffer (pH 6.0). Endogenous peroxidase activity was blocked with 3% hydrogen peroxide, followed by overnight incubation at 4°C with a primary anti-NCAN antibody (1:200; Affinity biosciences). The next day, sections were incubated with secondary antibody for 1 hour, developed using DAB, counterstained with hematoxylin, dehydrated, and mounted. Images were captured under a light microscope.

Quantitative assessment of NCAN immunostaining was performed using ImageJ software. Three to five randomly selected fields per section were analyzed to calculate the positive staining area (% Positive Staining Area) and integrated optical density (IOD), allowing for the comparison of NCAN expression between the two groups.

## 3 Results

### 3.1 Associations of plasma proteins with MASLD

**Figure 2:**
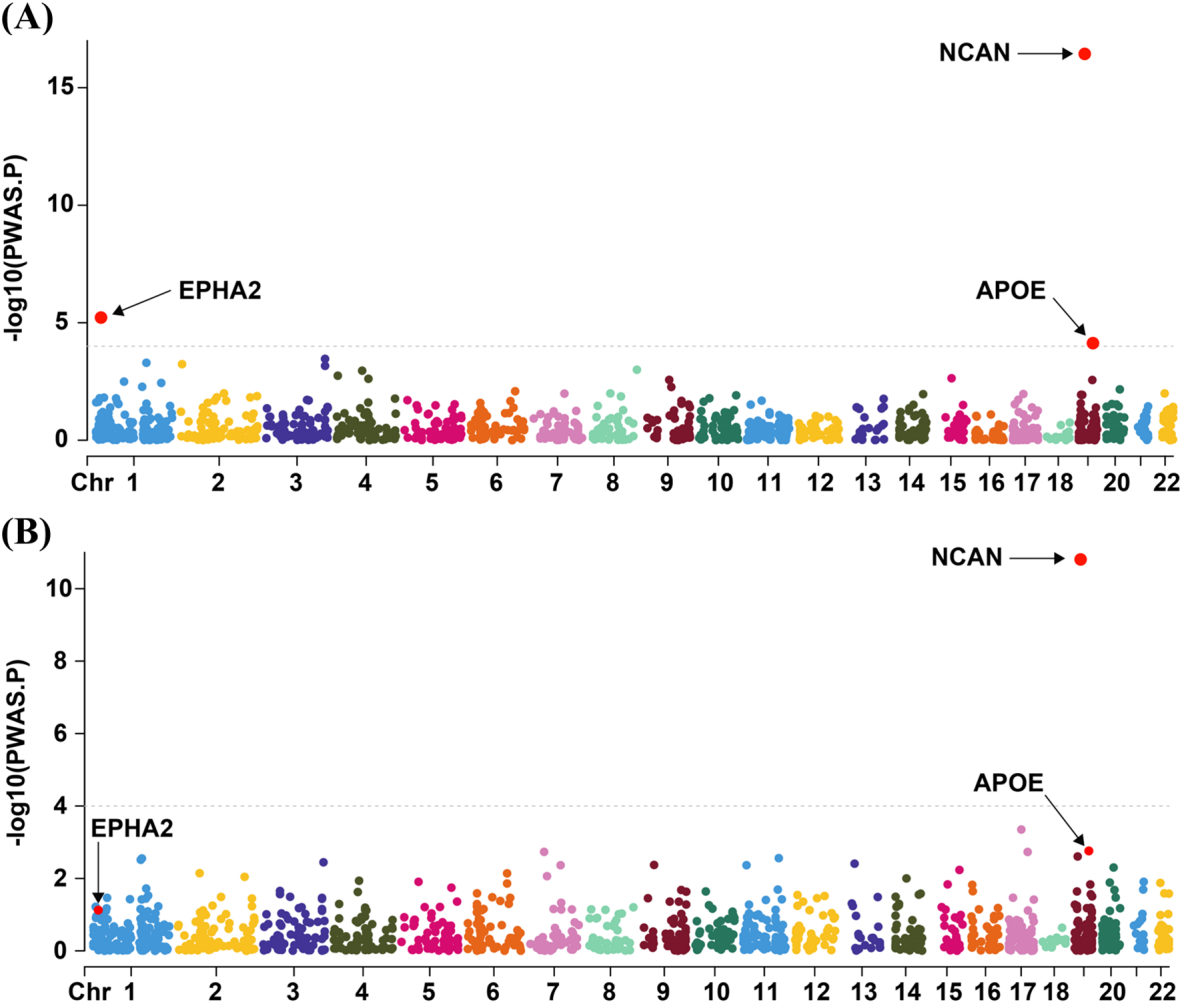
Visualization of PWAS Results for the discovery cohort and the validation cohort. The Manhattan plots summarizing the PWAS findings for the discovery cohort **(A)** and the validation cohort **(B)** are presented in panels A and B, respectively. Genes represented by red dots above the dashed line correspond to those surpassing the FDR threshold of 0.05. PWAS, proteome-wide association study; Chr, chromosome.

In the discovery cohort (R11 MASLD), we identified three genes (*NCAN*, *EPHA2*, and *APOE*) whose cis-regulated plasma protein levels were significantly associated with MASLD risk, as determined by PWAS (FDR < 0.05; **Figure 2A**; **Supplementary Table S1**). To further validate these associations, we performed a replication analysis using an independent MASLD GWAS dataset. In this validation analysis, only NCAN remained significantly associated with MASLD (FDR < 0.05; **Figure 2B**; **Supplementary Table S2**). Specifically, higher genetically predicted plasma levels of NCAN were consistently associated with a lower risk of MASLD in both cohorts (Z-score = -8.424, *p* = 3.64 × 10–^17^ in the discovery cohort; Z-score = -6.742, *p* = 1.56 × 10–^11^ in the validation cohort), as summarized in **Table 1**. By contrast, EPHA2 showed a significant inverse association with MASLD only in the discovery cohort (Z-score = -4.525, *p* = 6.04 × 10^-6^), but this association did not replicate in the validation analysis due to lack of statistical significance (**Table 1**). Similarly, APOE abundance predicted by the enet model was significantly associated with reduced MASLD risk in the discovery dataset (Z-score = -3.963, *p* = 7.41 × 10^-5^), yet failed to achieve significance in the validation cohort (**Table 1**). All associations in the discovery phase passed multiple testing correction (FDR < 0.05).

**Table 1:** Summary of PWAS-identified genes significantly associated with MASLD in the discovery and validation cohorts.

Genes	Z-score/OR/PP4	Discovery cohort			Validation cohort		
		Statistical estimates	P-value	FDR	Statistical estimates	P-value	FDR
NCAN	Z-score in PWAS	-8.424	3.64×10^-17^	4.82×10^-14^	-6.742	1.56×10^-11^	2.00×10^-8^
	OR (95% CI) in MR	0.441(0.328-0.593)	5.88×10^-8^	5.35×10^-6^	0.631(0.551-0.721)	1.56×10^-11^	1.20×10^-9^
	PP4 in colocalization	0.110	–	–	0.0002	–	–
EPHA2	Z-score in PWAS	-4.525	6.04×10^-6^	4.00×10^-3^	-1.779	7.52×10^-2^	7.51×10^-1^
	OR (95% CI) in MR	0.769(0.649-0.910)	2.25×10^-3^	2.28×10^-2^	–	–	–
	PP4 in colocalization	0.971	–	–	–	–	–
APOE	Z-score in PWAS	-3.963	7.41×10^-5^	3.27×10^-2^	-3.132	1.74×10^-3^	3.95×10^-1^
	OR (95% CI) in MR	0.730(0.253-2.105)	5.60×10^-1^	5.93×10^-1^	0.878(0.383-2.009)	7.58×10^-1^	5.83×10^-1^
	PP4 in colocalization	0.144	–	–	0.016	–	–

PWAS, proteome-wide association study; MASLD, metabolic dysfunction-associated steatotic liver disease; MR, Mendelian Randomization; OR, odds ratio; CI, confidence interval; FDR, false discovery rate; PP, posterior probability.

To elucidate the functional implications of genes identified through PWAS (*p* < 0.05), we performed GO and KEGG enrichment analyses. In the GO-BP category, the genes were predominantly enriched in pathways related to signal transduction, cell adhesion, inflammatory responses, and regulation of cell proliferation (**Supplementary Figure S1A**). These findings suggest that the associated proteins may influence MASLD development by modulating immune activation, intercellular communication, and tissue remodeling. In the GO-CC analysis, the genes were mainly localized to the plasma membrane, extracellular region, exosomes, and cell surface (**Supplementary Figure S1B**). The GO-MF terms were enriched for identical protein binding, calcium ion binding, and heparin binding (**Supplementary Figure S1C**). KEGG pathway analysis revealed that the identified genes were significantly involved in cytokine-cytokine receptor interactions and the JAK-STAT signaling cascade (**Supplementary Figure S1D**). These pathways are well-known regulators of immune function and inflammation. To further investigate the interplay among these proteins, we constructed a PPI network using the STRING database, which comprised 45 nodes and 57 edges (**Supplementary Figure S2A**). This network illustrates a coordinated regulatory architecture potentially underlying the observed genetic associations. Using the MCODE plugin in Cytoscape, three distinct modules with high intra-connectivity were identified within the network, with MCODE scores ≥3 and the top module scoring ≥4 (**Supplementary Figures S2B–D**). Additionally, key hub genes were identified using the Betweenness algorithm implemented in the cytoHubba tool. The top ten hubs—APOE, EFNB2, KNG1, NRP1, PF4, F10, EPHA2, GPT, PLXND1, and NLGN2—likely represent central regulators within the MASLD-related protein network (**Supplementary Figure S2E**).

### 3.2 Causal associations of identified proteins by PWAS with MASLD

**Figure 3:**
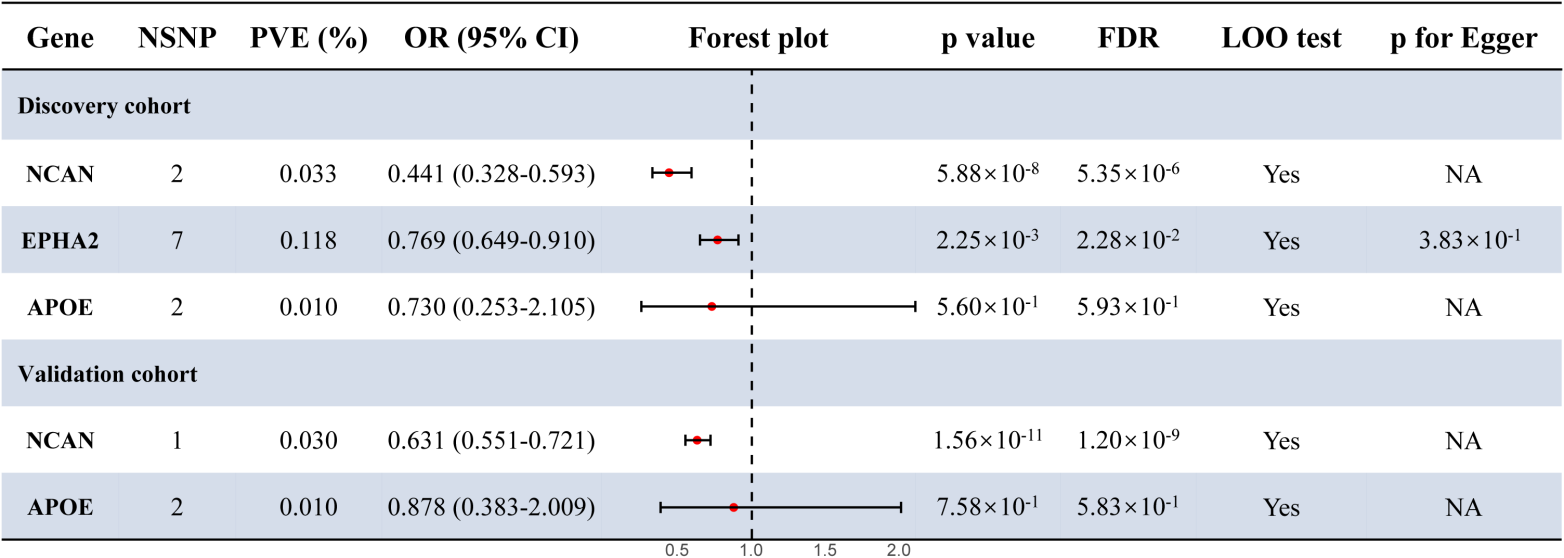
Forest plot for the MR results in the discovery cohort and the validation cohort. Estimates were derived using the IVW method. Statistical significance was defined as FDR < 0.05. *p* for Egger refers to the intercept *p*-value from MR-Egger regression, used to evaluate the presence of directional pleiotropy. NSNP, number of single nucleotide polymorphism; MR, Mendelian Randomization; OR, odds ratio; CI, confidence interval; PVE, proportion of variance explained; LOO, leave one out; NA, not available.

To investigate potential causal links between plasma proteins and susceptibility to MASLD, we extracted pQTLs and conducted MR analyses. In the case of the discovery cohort, the IVW method identified two (NCAN, EPHA2) out of three PWAS-prioritized genes as having statistically significant causal relationships with disease risk after multiple testing correction (FDR < 0.05; **Figure 3**; **Supplementary Tables S3–4**). In addition, in the validation cohort, MR analysis using the IVW method confirmed a significant causal association between NCAN and MASLD risk (FDR <0.05, **Figure 3**; **Supplementary Tables S3, S5**). EPHA2, however, lacked sufficient valid instrumental variables in this dataset and was therefore excluded from MR analysis.

As shown in **Figure 3** and **Table 1**, the NCAN gene demonstrated a consistent and statistically significant causal association with MASLD risk in both the discovery cohort (OR = 0.441, FDR = 5.35 × 10^-6^) and the validation cohort (OR = 0.631, FDR = 1.20 × 10^-9^). While EPHA2 was significantly associated with MASLD risk in the discovery cohort (OR = 0.769, FDR = 2.28 × 10^-2^), no valid instrumental variables were available for this gene in the validation cohort and it was therefore excluded from the corresponding MR analysis.

### 3.3 Shared causal variants of proteins and MASLD

**Figure 4:**
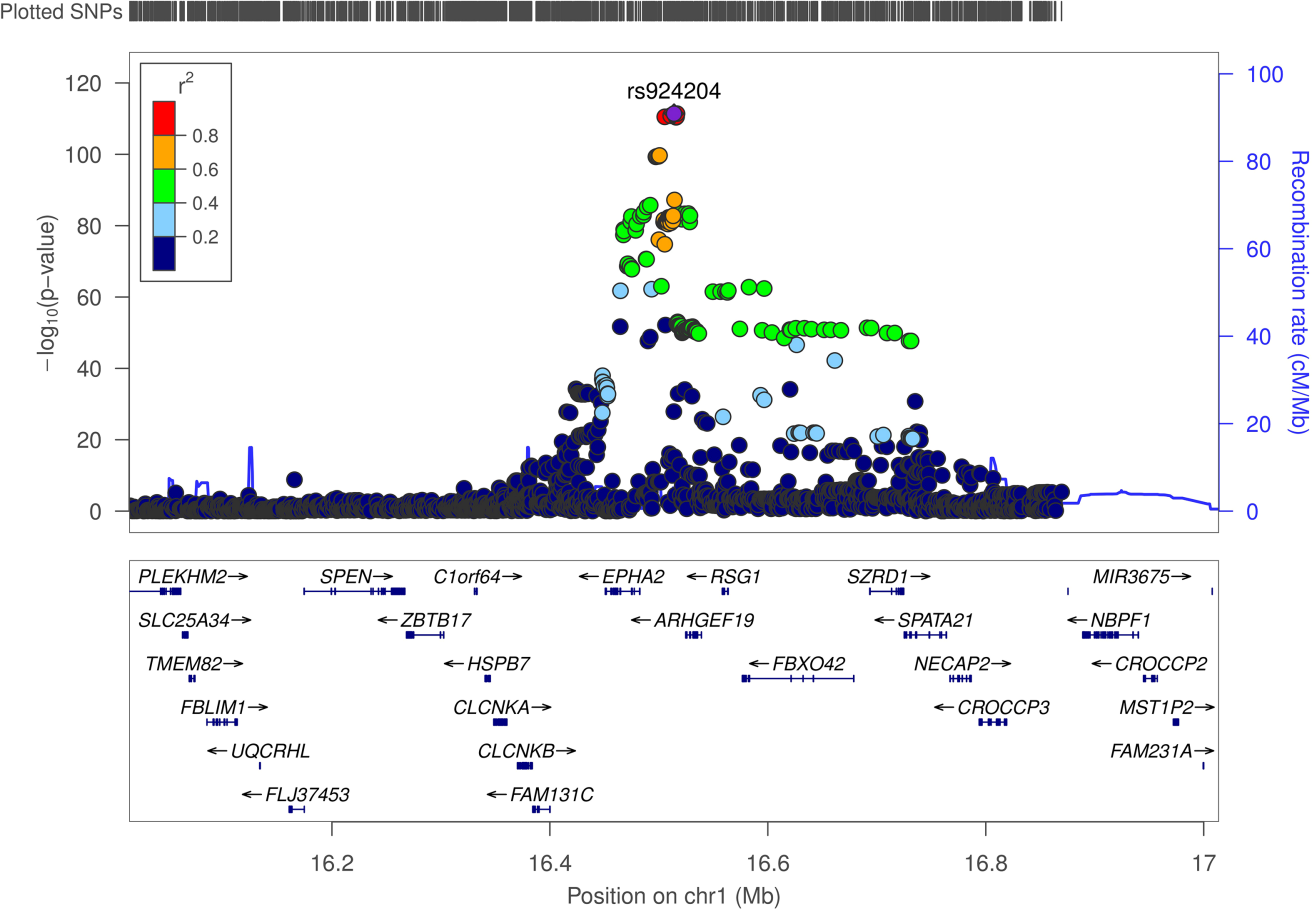
Illustration of the colocalization results in the discovery cohort. The significant colocalization results for the discovery cohort are presented, where regional association plots depict the alignment between genetic signals for MASLD risk and cis-pQTL associations at the *EPHA2* loci. Chr, chromosome.

To evaluate the likelihood that the same causal variant influences both protein expression and susceptibility to MASLD, Bayesian colocalization analyses were conducted for proteins identified as significant in the PWAS. Using PP4 ≥ 0.8 as the prespecified threshold, EPHA2 meets the colocalization criterion in the discovery cohort (PP4 = 0.971) (**Table 1**; **Figure 4**), supporting a shared causal variant at this locus. By contrast, NCAN (PP4 = 0.110 in discovery; 0.0002 in validation) and APOE (PP4 = 0.144 in discovery; 0.160 in validation) fall below the colocalization threshold; however, both loci showed high joint association probabilities (PP3+PP4 ≥ 0.8)—NCAN had PP3+PP4 = 1.000 in both datasets and APOE had PP3+PP4 = 0.992 in validation (**Supplementary Table S6**; **Supplementary Figure S3**)—indicating that each region harbors association signals for both traits even though a shared causal variant (PP4) is not supported at current power. We therefore interpret NCAN and APOE as suggestive, hypothesis-generating signals.

### 3.4 H&E staining and NCAN IHC results

**Figure 5:**
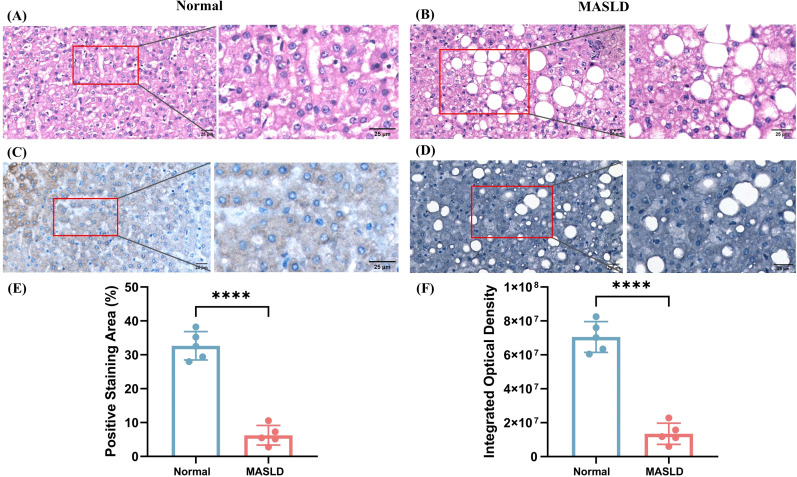
Histological and immunohistochemical analysis of liver tissues from normal and MASLD patients. **(A)** Representative H&E-stained section of normal liver showing intact hepatic architecture. **(B)** H&E staining of MASLD liver tissue demonstrating marked steatosis with numerous lipid droplets. **(C)** IHC for NCAN in normal liver tissue showing strong cytoplasmic staining. **(D)** IHC for NCAN in MASLD liver tissue indicating markedly reduced expression. **(E)** Quantification of the positive staining area (%) for NCAN in normal and MASLD tissues. **(F)** Quantification of IOD for NCAN in normal and MASLD tissues. ****P < 0.0001 versus the normal group.

H&E staining revealed that hepatocytes in normal liver tissues were arranged in a regular pattern with intact architecture, whereas MASLD tissues exhibited marked steatosis, ballooning degeneration, and inflammatory cell infiltration (**Figures 5A, B**). Immunohistochemical analysis showed moderate brown cytoplasmic staining of NCAN in normal liver tissues, while the NCAN-positive signal was markedly attenuated in MASLD tissues (**Figures 5C, D**), consistent with the proteomic findings. Semi-quantitative analysis further demonstrated that both the percentage of NCAN-positive staining area and the integrated optical density were significantly lower in MASLD tissues than in normal tissues (*p* < 0.01; **Figures 5E, F**; **Supplementary Table S7**), indicating a substantial downregulation of NCAN expression in MASLD. Sample information is provided in **Supplementary Table S8**.

## 4 Discussion

In this research, we integrated plasma protein pQTL data with GWAS results for MASLD to investigate potential functional relationships. Our findings from the R11 MASLD discovery cohort identified three significant proteins—NCAN, EPHA2, and APOE—whose genetically predicted plasma levels were associated with MASLD risk. Among these, NCAN demonstrated the strongest and most consistent signal, which was successfully replicated in the validation cohort. In contrast, the associations for EPHA2 and APOE did not reach significance in the validation analysis. Moreover, MR analyses provided additional causal support for the involvement of NCAN in both conditions. Bayesian colocalization analysis provided suggestive evidence of regional overlap at the *NCAN* locus for plasma protein levels and MASLD risk in both datasets. Taken together, these results strongly suggest that NCAN plays a causal and protective role in MASLD.

Given its consistent and robust associations across both the discovery cohort and the validation cohort, NCAN likely plays a pivotal role in the pathogenesis of hepatic injury. The *NCAN* locus spans approximately 500 kb on chromosome 19p13 and encompasses at least 20 genes (28). It encodes neurocan, a chondroitin sulfate proteoglycan primarily implicated in cell adhesion and migration within the nervous system (29, 30). Traditionally, NCAN was thought to influence metabolic regulation predominantly through its effects in the central nervous system (CNS), which plays a critical role in peripheral glucose and lipid homeostasis (31). However, subsequent studies by Nischalke et al. (32) revealed that *NCAN* is also expressed in hepatic tissue, suggesting a broader physiological relevance. Further research identified the SNP rs2228603 within *NCAN*, which results in a proline-to-serine substitution at position 92. This variant has been strongly associated with alterations in plasma low-density lipoprotein and triglyceride levels (33). Gorden et al. (29) also demonstrated that the rs2228603[T] allele constitutes a risk factor for hepatic inflammation and fibrosis, indicating its potential role in the progression from simple steatosis to steatohepatitis. However, MR analyses have not consistently confirmed a causal relationship. In our current study, we observed that higher plasma NCAN levels were causally associated with a reduced risk of MASLD, with the effect being driven by a *cis*-acting variant near *NCAN*. These findings highlight NCAN as a potentially important factor in the pathogenesis of MASLD. Nevertheless, contrasting evidence exists: for instance, Wu et al. (31) reported that *NCAN* rs2228603 was not a risk factor for MASLD incidence in the Chinese population. Moreover, they found that the T allele exhibits a dual role — providing hepatic protection by elevating high-density lipoprotein levels while simultaneously increasing the risk of liver damage via elevated alkaline phosphatase levels. Similarly, Lin et al. (34) demonstrated that *NCAN* was not a risk gene for MASLD in obese Taiwanese children. These discrepancies suggest a potential population-specific effect and underscore the need for further investigation into the context-dependent role of NCAN in MASLD development.

Moreover, our MR indicates that genetically proxied higher plasma NCAN associates with lower MASLD risk, whereas IHC shows reduced hepatic NCAN in MASLD. These findings are not mutually exclusive once compartment biology is considered. NCAN is a lectican-type chondroitin-sulfate proteoglycan embedded in the extracellular matrix (ECM) that undergoes proteolytic processing by ADAMTS family proteases, generating soluble fragments detectable in circulation (35, 36); by contrast, IHC predominantly reflects ECM-bound pools and is epitope-dependent. Accordingly, plasma and tissue readouts may track related but distinct proteoforms/compartments that move in the same direction yet differ in magnitude across disease stages. In addition, the SOMAmer-based platform quantifies epitope-specific proteoforms (37), whereas IHC may recognize a different epitope, reinforcing the need for proteoform-resolved validation. For these reasons, we avoid inferring that hepatic downregulation per se is causal in the same direction as the plasma association. Further experimental validation is warranted.

Our study found that EPHA2 in plasma is associated with a lower risk of MASLD. EPHA2, a member of the Eph receptor tyrosine kinase family, plays a crucial role in mediating cell–cell communication, and is involved in various biological processes including cell migration, proliferation, and angiogenesis (38). It is widely expressed in hepatic tissue and other organs (39). Consistent with our findings, Li et al. (40) recently identified *EPHA2* as a candidate gene for MASLD susceptibility through an enhancer-gene regulatory map of the liver, highlighting non-coding SNPs located in active regulatory regions that are linked to *EPHA2* expression. However, in contrast to our observations, Pearson-Gallion et al. (41) reported that *EphA2* knockout mice fed a high-fat diet developed significantly less hepatic steatosis and inflammation than wild-type controls, suggesting that excessive or dysregulated EPHA2 signaling may exacerbate MASLD progression. Notably, our findings revealed an inverse association between circulating EPHA2 protein levels and MASLD risk, which appears contradictory to the pro-steatotic role observed in preclinical models. This discrepancy may be attributed to the context-dependent and compartment-specific function of EPHA2: while tissue-localized, ligand-independent activation of EPHA2 may promote hepatic inflammation and fibrosis, higher levels of EPHA2 in circulation could reflect a compensatory or protective systemic signaling state, or alternatively, reduced hepatic EPHA2 activation. Moreover, plasma EPHA2 may serve as a biomarker for favorable metabolic or immune status rather than a direct mediator of disease risk. These observations underscore the complexity of EPHA2’s role in liver pathophysiology and highlight the need for further studies to dissect its tissue-specific functions and regulatory mechanisms in MASLD.

Our study found that plasma APOE levels are associated with a lower risk of MASLD, suggesting a potentially protective role of APOE in regulating hepatic lipid accumulation. APOE is a liver-derived apolipoprotein involved in both the assembly and clearance of very low-density lipoproteins, playing a key role in maintaining plasma triglyceride levels and lipid homeostasis (42). Beyond its role in lipid metabolism, APOE also exhibits anti-inflammatory and antioxidant properties, which may contribute to liver health through non-lipid-related mechanisms (43). Consistent with our findings, multiple studies in *ApoE^−/−^* mice have demonstrated that the absence of APOE exacerbates MASLD progression, particularly under high-fat diet conditions (43–46). The inverse association observed in our study between circulating APOE levels and MASLD risk may therefore reflect APOE’s protective functions in maintaining hepatic lipid balance and mitigating liver injury. Notably, MASLD is a heterogeneous, multi-factorial disorder, so a single protein is unlikely to constitute a stand-alone therapy. Our genetics-anchored signals (e.g., NCAN, EPHA2, APOE) should be viewed as hypothesis-generating candidates rather than established targets. Concurrently, work by Moliterni et al. (47) also supports a pathway- and network-level interpretation of dyslipidaemia. Finally, because hepatic lipid handling and inflammatory tone are under circadian control, time-of-day effects may also modulate circulating proteins and MASLD biology (48, 49).

This study possesses several notable strengths alongside certain limitations. One major advantage lies in the application of PWAS methods to identify novel protein targets related to MASLD, utilizing the most extensive and detailed plasma pQTL dataset available, alongside GWAS data encompassing approximately 500,000 individuals. Furthermore, MR analysis was conducted to strengthen the inference of causality between the identified proteins and MASLD. To further evaluate whether shared causal variants underlie both protein expression and disease phenotypes, Bayesian colocalization analysis was employed, ultimately confirming NCAN as a likely pathogenic factor for MASLD. Collectively, these rigorous approaches enhance the robustness and reliability of our findings.

Nevertheless, several limitations should be acknowledged. First, although the pQTL study profiled 4,483 plasma proteins, the SOMAmer-based detection platform does not capture the entirety of the plasma proteome. As a result, proteins not covered by this assay may also contribute to MASLD susceptibility and progression, but remain unexamined in this study. Second, our analyses were restricted to individuals of European ancestry. While this design minimizes potential confounding due to population stratification, it also restricts the applicability of the results to other ethnic groups. Future research involving diverse populations and larger sample sizes will be essential to validate and extend these findings. Third, case ascertainment relied on administrative ICD codes rather than uniform imaging, biopsy, or laboratory criteria across cohorts. This approach enables large-scale analyses but can introduce residual phenotype misclassification and cross-cohort heterogeneity, potentially attenuating true associations or inflating uncertainty. Lastly, Changes in plasma protein levels may reflect compensatory responses, shedding/clearance dynamics, tissue redistribution, or disease activity and therefore do not, by themselves, establish druggability or causality. Accordingly, our protein findings are hypothesis-generating and require follow-up validation, such as pathway-level mechanistic studies in liver-relevant cell and animal models.

## 5 Conclusion

In summary, this study identified three plasma proteins—NCAN, EPHA2, and APOE—that were significantly associated with MASLD risk in the discovery cohort. Among them, NCAN demonstrated the strongest and most consistent evidence, with replication in an independent validation dataset. MR analyses supported a causal relationship between NCAN and MASLD risk, and Bayesian colocalization further provided suggestive regional overlap linking plasma NCAN protein levels to disease susceptibility. These findings highlight NCAN as a promising therapeutic target for MASLD.

## Data Availability

The GWAS data for the discovery cohort were retrieved from the FinnGen R11 dataset (https://r11.finngen.fi/pheno/NAFLD). The GWAS data for the validation cohort were obtained from the largest published GWAS meta-analysis of MASLD to date[19]. All analysis scripts (PWAS, MR, and colocalization) and derived summary tables are deposited at Zenodo under DOI: 10.5281/zenodo.17202121. No individual-level data are shared. Figure source data and README documentation are included in the Zenodo record.
