# Supplementary material for: A proteome-wide association study reveals novel plasma proteins as potential therapeutic targets for metabolic dysfunction-associated steatotic liver disease
Source: Front Endocrinol (Lausanne). 2025 Oct 16;16:1664691. doi: 10.3389/fendo.2025.1664691 (PMC12571593; doi:10.3389/fendo.2025.1664691)
Supplement: Supplementary file 1 [file DataSheet1.docx]

**Supplemental materials**

1. **Table S1: Significant genes associated with MASLD in the discovery cohort identified through PWAS** (**Page 2**).
2. **Table S2: Significant genes associated with MASLD in the validation cohort identified through PWAS (Page 3)**.
3. **Table S3: IVs in the discovery and validation cohorts (Page 4).**
4. **Table S4: MR results of MASLD in the discovery cohort using IVW and MR-Egger methods (Page 5)**.
5. **Table S5: MR results of MASLD in the validation cohort using IVW and MR-Egger methods** (**Page 6**).
6. **Table S6: Bayesian colocalization results for proteins associated with MASLD in the discovery and validation datasets (Page 7)**.
7. **Table S7: Quantitative analysis of NCAN immunostaining in liver tissues from normal and MASLD groups (Page 8).**
8. **Table S8: Participant characteristics of the liver cohort (Page 9).**
9. **Figure S1: Results of GO and KEGG analyses** (**Page 10**).
10. **Figure S2: Protein interaction network and key modules** (**Page 11**).
11. **Figure S3: Illustration of the colocalization results in the validation cohorts** (**Page 12**).

| **Table S1: Significant genes associated with MASLD in the discovery cohort identified through PWAS.** | | | | | | | |
| --- | --- | --- | --- | --- | --- | --- | --- |
| **ID** | **Chr** | **NSNP** | **Model** | **MODELCV.R2** | **PWAS Z-score** | **PWAS P value** | **FDR** |
| **NCAN** | 19 | 24 | top1 | 0.03 | -8.424 | 3.64E-17 | 4.82E-14 |
| **EPHA2** | 1 | 49 | enet | 0.094 | -4.525 | 6.04E-06 | 4.00E-3 |
| **APOE** | 19 | 17 | enet | 0.003 | -3.963 | 7.41E-05 | 3.27E-2 |

PWAS: proteome-wide association study; MASLD: metabolic dysfunction-associated steatotic liver disease; FDR: false discovery rate; NSNP: number of single nucleotide polymorphism; Chr: chromosome.

| **Table S2: Significant genes associated with MASLD in the validation cohort identified through PWAS.** | | | | | | | |
| --- | --- | --- | --- | --- | --- | --- | --- |
| **ID** | **Chr** | **NSNP** | **Model** | **MODELCV.R2** | **PWAS Z-score** | **PWAS P value** | **FDR** |
| **NCAN** | 19 | 24 | top1 | 0.03 | -6.742 | 1.56E-11 | 2.00E-08 |
| **EPHA2** | 1 | 49 | enet | 0.094 | -1.779 | 7.52E-02 | 7.51E-01 |
| **APOE** | 19 | 17 | enet | 0.003 | -3.132 | 1.74E-03 | 3.95E-01 |

PWAS: proteome-wide association study; MASLD: metabolic dysfunction-associated steatotic liver disease; FDR: false discovery rate; NSNP: number of single nucleotide polymorphism; Chr: chromosome.

| **Table S3: IVs in the discovery and validation cohorts.** | | | | | | | | | | |
| --- | --- | --- | --- | --- | --- | --- | --- | --- | --- | --- |
| **Proteins** | **SNP** | **CHR** | **BP** | **A1** | **A2** | **beta.exposure** | **se.exposure** | **beta.outcome** | **se.outcome** | **F statistics** |
| IVs in R11_MASLD | | | | | | | | | | |
| APOE | rs35136575 | 19 | 45439163 | G | C | -0.134428 | 0.0195404 | -0.0133655 | 0.0297869 | 47.32739224 |
|  | rs75627662 | 19 | 45413576 | T | C | 0.111927 | 0.0209687 | -0.114171 | 0.0323497 | 28.4922479 |
| NCAN | rs2228603 | 19 | 19329924 | T | C | -0.451966 | 0.030094 | 0.38382 | 0.045565 | 225.5546047 |
|  | rs75072017 | 19 | 19344059 | G | A | 0.226369 | 0.049292 | -0.0139798 | 0.114419 | 21.09021587 |
| EPHA2 |  |  |  |  |  |  |  |  |  |  |
|  | rs10927888 | 1 | 16353511 | T | C | -0.22864 | 0.0446075 | 0.192898 | 0.0854179 | 26.27172894 |
|  | rs115041526 | 1 | 16417553 | T | C | -0.30661 | 0.0666872 | -0.088987 | 0.0744079 | 21.139157 |
|  | rs12401740 | 1 | 16056622 | T | G | 0.292854 | 0.0652646 | 0.0694821 | 0.0869718 | 20.13478328 |
|  | rs2273312 | 1 | 16542937 | G | T | 0.0967906 | 0.0200468 | -0.0624874 | 0.0315993 | 23.31182364 |
|  | rs34192549 | 1 | 16464489 | T | C | 0.907309 | 0.0590648 | -0.197345 | 0.124844 | 235.9679189 |
|  | rs532881410 | 1 | 16830127 | T | C | 0.609788 | 0.0791222 | -0.128545 | 0.16306 | 59.39652518 |
|  | rs924204 | 1 | 16513926 | A | G | 0.371275 | 0.0162093 | -0.120308 | 0.0283366 | 524.6418045 |
| IVs in MASLD | | | | | | | | | | |
| APOE | rs35136575 | 19 | 45439163 | G | C | -0.134428 | 0.0195404 | -0.0279053 | 0.0195076 | 47.32739224 |
|  | rs75627662 | 19 | 45413576 | T | C | 0.111927 | 0.0209687 | -0.0737329 | 0.0203042 | 28.4922479 |
| NCAN | rs2228603 | 19 | 19329924 | T | C | -0.451966 | 0.030094 | 0.2084325 | 0.030915 | 225.5546047 |

IVs, instrumental variables; SNP, single-nucleotide polymorphism; CHR, chromosome; BP, base-pair position (genome build per Methods); A1, effect allele aligned to the exposure (protein); A2, non-effect/other allele; β_exposure (beta_exposure), per-allele effect of the SNP on plasma protein levels (units as defined in Methods); SE_exposure (se_exposure), standard error of β_exposure; β_outcome (beta_outcome), per-allele log-odds (or effect) on MASLD risk; SE_outcome (se_outcome), standard error of β_outcome; F-statistics, first-stage instrument strength (per-SNP F); MASLD, metabolic dysfunction-associated steatotic liver disease; R11, FinnGen Release 11 (discovery cohort).

| **Table S4: MR results of MASLD in the discovery cohort using IVW and MR-Egger methods.** | | | | | | | | | | | | |
| --- | --- | --- | --- | --- | --- | --- | --- | --- | --- | --- | --- | --- |
| **Gene** | **Chr** | **NSNP** | **PVE**  **(%)** | **Loo**  **test** | **IVW estimator** | | | **MR-Egger estimator** | | | **P for**  **Egger intercept** | **P for heterogeneity (IVW)** |
|  |  |  |  |  | **OR (95CI)** | **P value** | **FDR** | **OR (95CI)** | **P value** | **FDR** |  |  |
| NCAN | 19 | 2 | 0.033 | Yes | 0.441(0.328-0.593) | 5.88E-8 | 5.35E-6 | NA | NA | NA | NA | NA |
| EPHA2 | 1 | 7 | 0.118 | Yes | 0.769(0.649-0.910) | 2.25E-3 | 2.28E-2 | 0.858(0.608-1.211) | 3.83E-01 | 7.44E-01 | 4.68E-01 |  |
| APOE | 19 | 2 | 0.010 | Yes | 0.730(0.253-2.105) | 5.60E-1 | 5.93E-1 | NA | NA | NA | NA | NA |

MASLD: metabolic dysfunction-associated steatotic liver disease; MR: Mendelian Randomization; IVW: inverse variance weighting; FDR: false discovery rate; NSNP: number of single nucleotide polymorphism; Chr: chromosome; OR: odds ratio; CI: confidence interval; PVE: proportion of variance explained; LOO: leave one out; NA: not available.

| **Table S5: MR results of MASLD in the validation cohort using IVW and MR-Egger methods.** | | | | | | | | | | | | |
| --- | --- | --- | --- | --- | --- | --- | --- | --- | --- | --- | --- | --- |
| **Gene** | **Chr** | **NSNP** | **PVE**  **(%)** | **Loo**  **test** | **IVW** **estimator** | | | **MR-Egger estimator** | | | **P for**  **Egger intercept** | **P for heterogeneity (IVW)** |
|  |  |  |  |  | **OR (95CI)** | **P value** | **FDR** | **OR (95CI)** | **P value** | **FDR** |  |  |
| NCAN | 19 | 1 | 0.030 | Yes | 0.631(0.551-0.721) | 1.56E-11 | 1.20E-9 | NA | NA | NA | NA | NA |
| APOE | 19 | 2 | 0.010 | Yes | 0.878(0.383-2.009) | 7.58E-1 | 5.83E-1 | NA | NA | NA | NA | NA |

MASLD: metabolic dysfunction-associated steatotic liver disease; MR: Mendelian Randomization; IVW: inverse variance weighting; FDR: false discovery rate; NSNP: number of single nucleotide polymorphism; Chr: chromosome; OR: odds ratio; CI: confidence interval; PVE: proportion of variance explained; LOO: leave one out; NA: not available.

| **Table S6: Bayesian colocalization results for proteins associated with MASLD in the discovery and validation datasets.** | | | | | | | | | | |
| --- | --- | --- | --- | --- | --- | --- | --- | --- | --- | --- |
| **Gene** | **Causal SNP** | **NSNP** | **PP0** | **PP1** | **PP2** | **PP3** | **PP4** | **PP4+PP3** | **Causal** | **Trait** |
| NCAN | rs2228603 | 2034 | 4.74E-57 | 1.11E-14 | 3.80E-43 | 0.890339 | 0.109661 | 1.000 | FALSE | Discovery |
| EPHA2 | rs924204 | 2440 | 1.95E-110 | 0.020258277 | 9.28E-111 | 0.00868 | 0.971062 | 0.980 | TRUE | Discovery |
| APOE | rs5117 | 3075 | 3.32E-06 | 0.54922502 | 1.86E-06 | 0.307181 | 0.143589 | 0.451 | FALSE | Discovery |
| NCAN | rs2228603 | 1690 | 1.09E-54 | 2.55E-12 | 4.27E-43 | 0.999829 | 0.000171 | 1.000 | FALSE | Validation |
| APOE | rs429358 | 2585 | 5.05E-08 | 0.008332265 | 5.92E-06 | 0.97609 | 0.015571 | 0.992 | FALSE | Validation |

MASLD: metabolic dysfunction-associated steatotic liver disease; FDR: false discovery rate; NSNP: number of single nucleotide polymorphism; PP: posterior probability.

**Table S7: Quantitative analysis of NCAN immunostaining in liver tissues from normal and MASLD groups.**

| **Group** | **Image** | **Area** | **% Positive area** | **IOD (IntDen)** |
| --- | --- | --- | --- | --- |
| Normal | N1 | 846888 | 32.521 | 70230315 |
| Normal | N2 | 323787 | 38.233 | 82565685 |
| Normal | N3 | 236977 | 27.982 | 60429135 |
| Normal | N4 | 248813 | 29.380 | 63447315 |
| Normal | N5 | 298225 | 35.214 | 76047375 |
| MASLD | M1 | 846888 | 7.287 | 15736560 |
| MASLD | M2 | 89347 | 10.550 | 22783485 |
| MASLD | M3 | 43623 | 5.151 | 11123865 |
| MASLD | M4 | 46235 | 5.459 | 11789925 |
| MASLD | M5 | 23369 | 2.759 | 5959095 |

MASLD: metabolic dysfunction-associated steatotic liver disease; IOD: integrated optical density.

**Table S8: Participant characteristics of the liver cohort.**

| **Parameter** | **Controls (n=5)** | **MASLD cases (n=5)** |
| --- | --- | --- |
| Age, years (mean ± SD) | 52.38±8.324 | 54.29±9.137 |
| Sex, male/female, n | 2/3 | 2/3 |
| BMI, kg/m^2^ (mean ± SD) | 23.54±1.065 | 25.87±1.072 |
| Diabetes mellitus, n (%) | 0 (0%) | 5 (100%) |
| Total cholesterol, mmol/L (mean ± SD) | 3.443±0.2256 | 4.417±0.1341 |
| Triglycerides, mmol/L (mean ± SD) | 1.247±0.08192 | 1.479±0.1201 |
| HDL-C, mmol/L (mean ± SD) | 1.459±0.9831 | 1.185±0.1005 |
| LDL-C, mmol/L (mean ± SD) | 2.8157±0.2209 | 3.3671±0.2027 |
| Fibrosis stage (F0-F4), n | F0, 5 | F1, 1; F2, 4 |
| Medications n (%) | 0 (0%) | 5 (100%) |

MASLD, metabolic dysfunction-associated steatotic liver disease; SD, standard deviation; BMI, body mass index; HDL-C, high-density lipoprotein cholesterol; LDL-C, low-density lipoprotein cholesterol; n, number of participants; F0-F4, histological fibrosis stages (F0 = no fibrosis; F1 = mild/perisinusoidal or portal fibrosis; F2 = perisinusoidal and portal/periportal fibrosis; F3 = bridging fibrosis; F4 = cirrhosis) according to the Kleiner/Brunt system; Medications, baseline use of lipid-lowering or antidiabetic agents at biopsy.


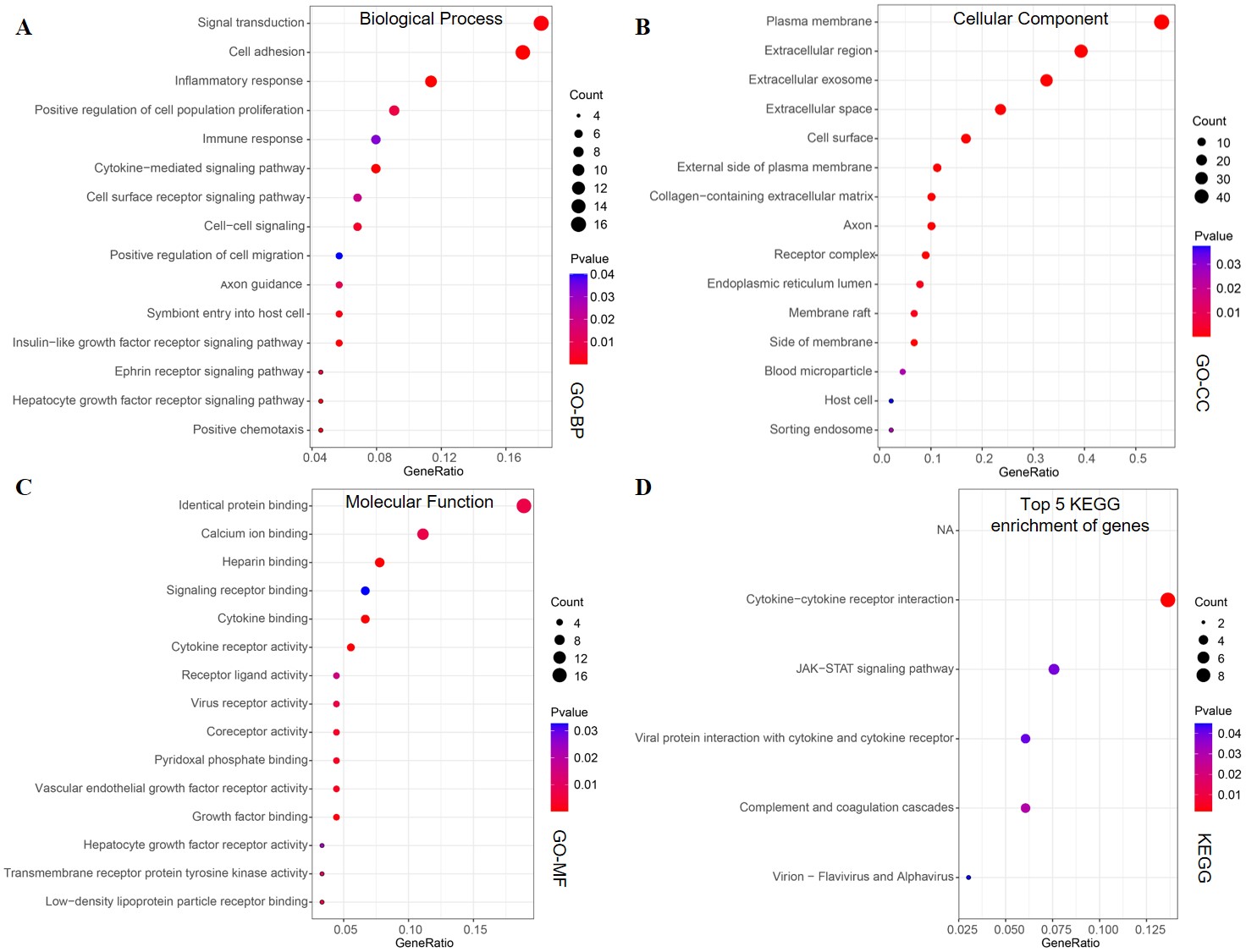


**Figure S1: Results of GO and KEGG analyses. A: GO-BP; B: GO-CC; C: GO-MF; D: KEGG.**

GO: gene ontology; KEGG: Kyoto Encyclopedia of Genes and Genomes; BP: biological process; CC: cellular component; MF: molecular function.


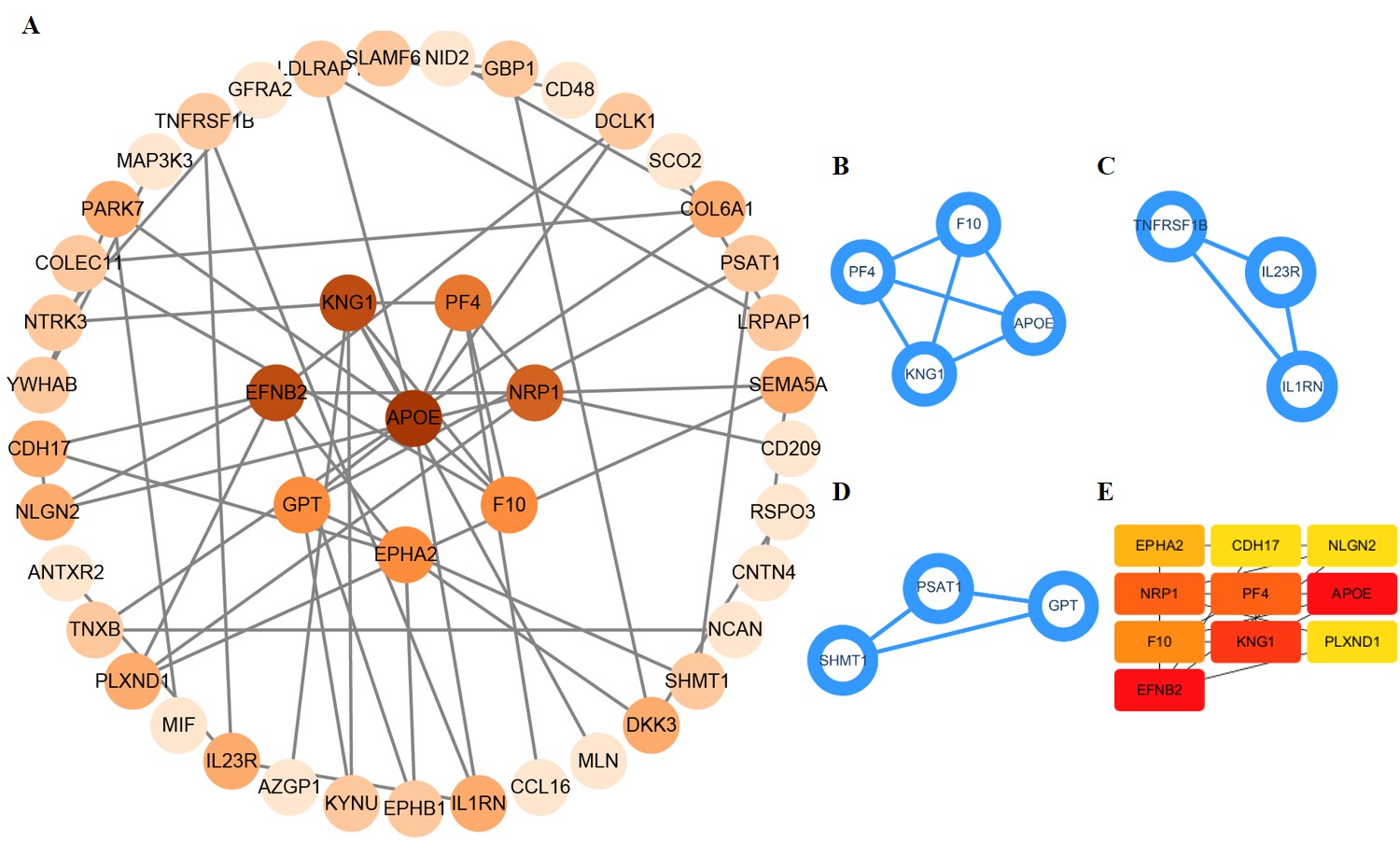


**Figure S2: Protein interaction network and key modules.** To explore functional associations among proteins, interaction data were obtained from the STRING database (https://string-db.org) and visualized using Cytoscape software (version 3.10.0). Key functional clusters within the PPI network were identified using the MCODE plugin, applying its default settings to detect densely connected regions. Additionally, the cytoHubba plugin was employed to rank and extract the top 20 hub genes based on their centrality scores within the network.


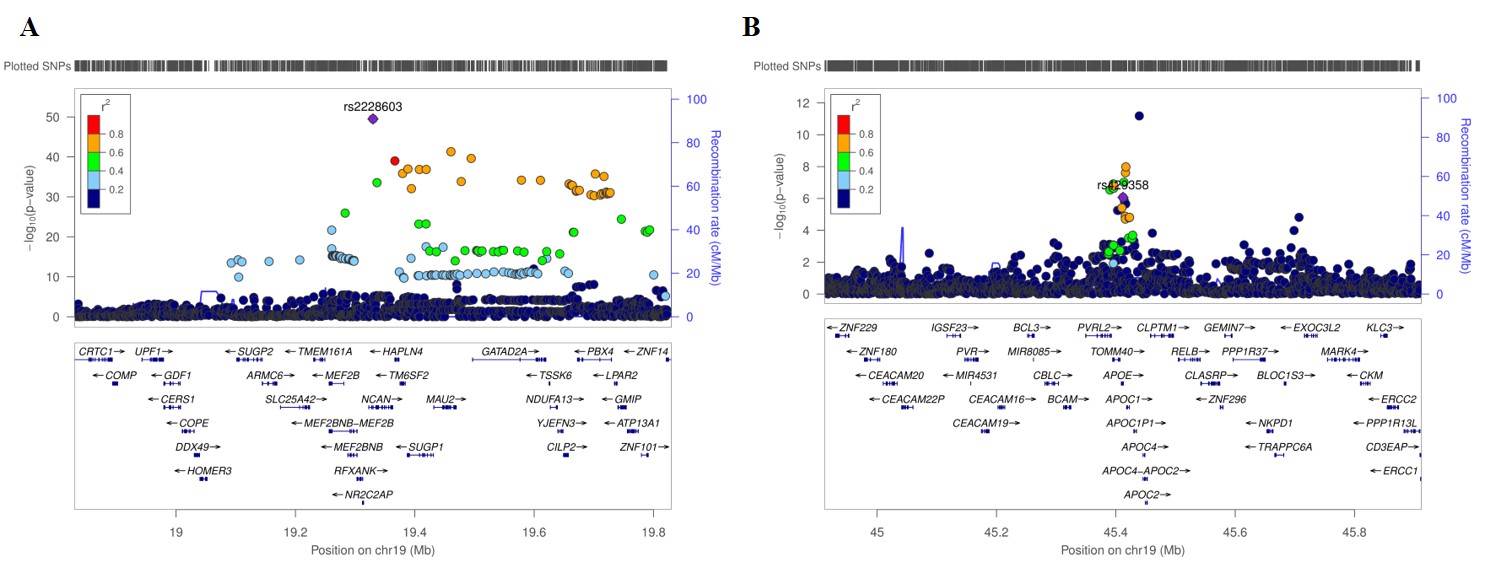


**Figure S3: Illustration of the colocalization results in the** **validation cohorts.** A. NCAN; B. APOE.
